# Novel uses of ensiled biomasses as feedstocks for green biorefineries

**DOI:** 10.1186/s40104-024-00992-y

**Published:** 2024-03-05

**Authors:** Marketta Rinne

**Affiliations:** Natural Resources Institute Finland (Luke), Jokioinen, Finland

**Keywords:** Additive, Fermentation, Liquid-solid separation, Protein, Silage

## Abstract

Perennial forage plants are efficient utilizers of solar radiation and nutrients so that there is a lot of scope to increase the production of green biomass in many areas. Currently, grasses are mainly used as feeds for ruminants and equines, but there could be higher added value use for several components of the green biomass. Interest in green biorefining has risen recently motivated by the increased sustainability pressures and need to break the reliance on fossil fuels. Novel products derived from grass, such as paper and packaging, nanofibers, animal bedding, novel protein feeds, extracted proteins, biochemicals, nutraceuticals, bioactive compounds, biogas and biochar could create new sustainable business opportunities in rural areas. Most green biorefinery concepts focus on using fresh green biomass as the feedstock, but preservation of it by ensiling would provide several benefits such as all-year-around availability of the feedstock and increased stability of the press juice and press cake. The major difference between fresh and ensiled grass is the conversion of water soluble carbohydrates into fermentation end products, mainly lactic and acetic acids, that lower the pH of the silage so that it becomes stable in anaerobic conditions. This has some important consequences on the processability and quality of products, which are partly positive and partly negative, e.g., degradation of protein into peptides, amino acids and ammonia. These aspects are discussed in this review.

**Graphical Abstract:**

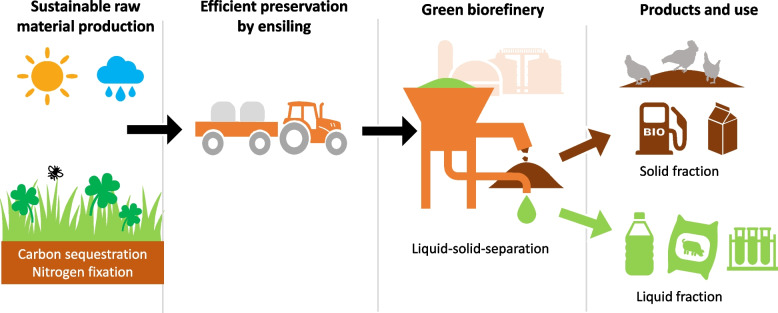

## Introduction

**Fig. 1:**
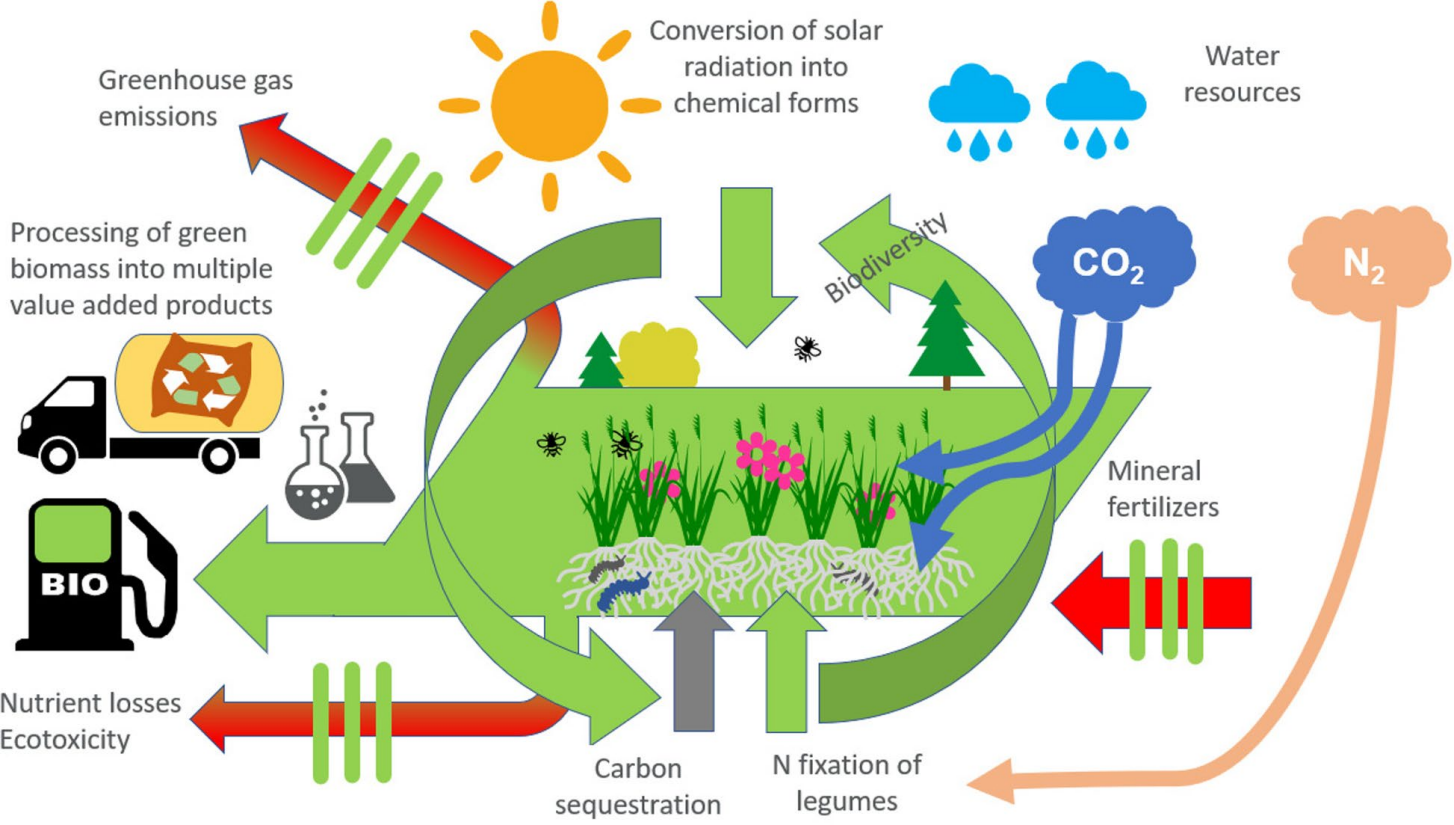
Increased utilization of green biomass can provide many ecosystem services

Humankind is becoming increasingly aware of the planetary boundaries and novel more sustainable ways to utilize natural resources are needed. Perennial forage plants are efficient utilizers of solar radiation and nutrients so that there is a lot of scope to increase the production of green biomass in many areas [1] and benefit from the ecosystem services grasslands can provide (Fig. 1). As an example of the biomass production potential, under the growing conditions of Finland, a two-fold dry matter (DM) yield for grasses is achieved compared to barley and oats grains [2].

Currently, grasses are mainly used as feeds for ruminants and equines, and to some extent in biogas production [3, 4], but there could be higher added value use for several components of the green biomass. Novel sustainable products derived from grass, such as paper and packaging, nanofibers, animal bedding, novel protein feeds, extracted proteins, biochemicals, nutraceuticals, bioactive compounds, biogas and biochar are seen to create new sustainable business opportunities in rural areas [5, 6]. The concept of biorefining green biomass is not novel, as pointed out in a recent review by Domokos-Szabolcsy et al. [7], who referred to the extensive work and number of publications by a Hungarian scientist and innovator Károly (Karl) Ereky since 1920’s. The use of protein extracted from green leaves for nutrition of humans and monogastric farm animals was also brought up in UK, motivated by the Second World War [8] and green crop fractionation was extensively studied in the 1970’s [9]. In the late 1900’s the global trade of good quality affordable soy protein however outcompeted green protein production. New interest in green biorefining has risen since the beginning of the 21st century, motivated by the increased sustainability pressures and need to break the reliance on fossil fuels [1, 6].

It is noteworthy that in the early green biorefining activities, fresh grass was used as the feedstock, and preservation by ensiling was not considered. The benefits of having a stable feedstock available all year around are obvious for green biorefineries as for livestock operations, and indeed ensiled materials have been in focus, e.g., in Austrian [10], Irish [11] and Finnish [12] approaches. It is essential that those involved in the biorefinery activities utilize the vast amount of knowledge gained in grassland and livestock sciences about how grassland management affects the yield and composition of the green biomass, and how preservation techniques affect the composition and losses, as these factors have important environmental and economic impacts. This review focuses on aspects related to the use of ensiled grass as the feedstock for green biorefineries.

### Multiple options for green biorefinery processes and products

**Fig. 2:**
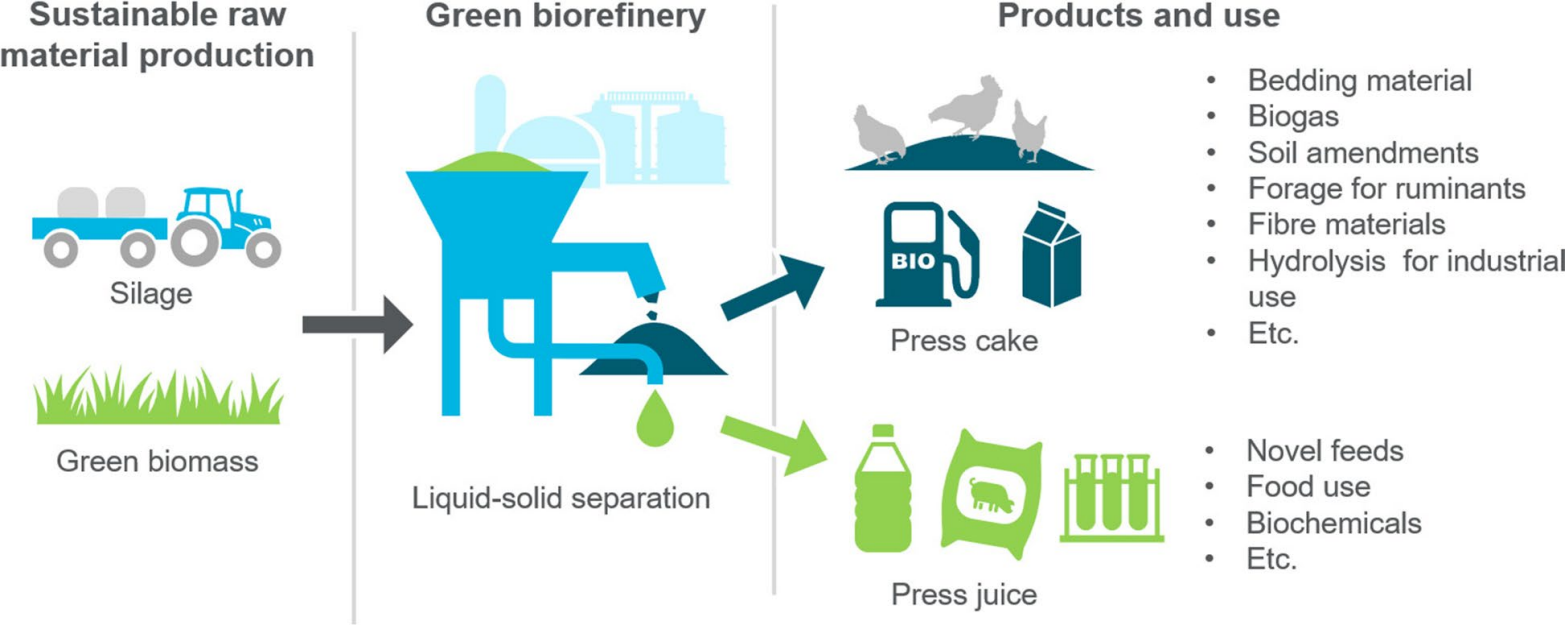
Schematic presentation of a green biorefinery approach (based on [13])

A simplified presentation of a green biorefinery concept is provided in Fig. 2. It shows that green biomass is first separated into liquid and solid fractions, and they are further processed to a range of products. The terminology varies to some extent so that liquid fraction can be called press juice or grass/silage juice, and for the solid fraction terms press cake and pulp have been used. In the current article, terms press juice and press cake are used.

Green biorefining as such is a very broad term that can include numerous different types of processes resulting in variable products [14]. This also means that the requirements for the raw materials are not constant. In addition, grass silage contains several components such as neutral detergent fibre, crude protein (CP), water soluble carbohydrates, fermentation end products, minerals and bioactive compounds to a variable extent, so that a single component cannot dominate the product portfolio. The number of various phytochemicals in green biomasses is high as demonstrated by the analytical work reported by Domokos-Szabolcsy et al. [7], who listed 46 different compounds identified by UHPLC-ESI-MS analysis. Potentially, extraction of phytochemicals of high added value could be included in a biorefinery operation.

This review concentrates on green biomasses, mainly grasses, that have been ensiled, but biorefining could be used for many different types of raw materials and by-products as well. High-moisture materials are produced in large quantities in several types of food industries, e.g., vegetable processing and beverage production, and use of the side streams could be improved by liquid-solid separation and further processing [15]. The process needs to be carefully thought through to achieve effective use of all the products of fractionation and to cover the increased costs caused by further processing.

There is much interest to use protein [6, 16] or even the fibre fractions [17] of the green biomass as human food components, but currently commercial applications are scarce. The development of food products is, at least within Europe, limited due to the novel foods act that requires authorization of novel food materials prior to the release to markets [18]. Thus, applications for feed, energy and materials use seem closer to reality at least in short to medium time span.

### Fresh vs. preserved grass for a biorefinery

The major difference between fresh and ensiled grass is the conversion of water soluble carbohydrates into fermentation end products, mainly lactic and acetic acids, that lower the pH of the silage so that it becomes stable in anaerobic conditions [19]. Ethanol, propionic and butyric acids and other minor fermentation end products are also formed, but typically in rather small concentrations. The extent of water soluble carbohydrate conversion to fermentation end products varies a lot and can be manipulated by management factors such as wilting and use of additives [19].

Ensiling fresh biomasses has become the mainstream technology in ruminant livestock operations [20] due to efficient logistics and good stability. These benefits would also apply in green biorefineries. However, ensiling has also some disadvantages and some of them, such as difficulty of extracting the protein from the press juice, and altered taste and smell (particularly for food applications) are specific to green biorefineries (Table 1).

**Table 1:** Synthesis of benefits and disadvantages of ensiling from the point of view of green biorefining

Benefits of ensiling	Disadvantages of ensiling
Stable raw material for year-around operation	Fermentation losses and potentially reduced hygienic quality
Improved stability of fractions	Degradation of protein and impaired possibilities to separate it
Established technology, contractors available in many places	Altered taste and smell

**Fig. 3:**
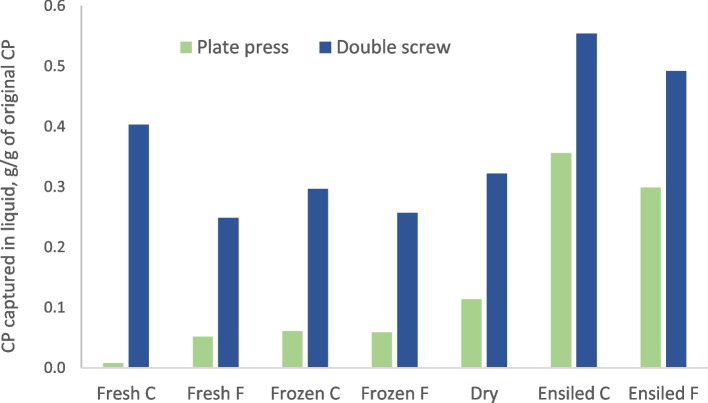
Comparison of crude protein extraction efficiency into press juice of fresh and preserved (frozen, dried and rehydrated or ensiled) without (C) or with a formic acid-based additive (F) using an inefficient plate press or an efficient double screw press [22]

Ensiling modifies the plant structure both physically (chopping, compacting) and chemically. Some acid hydrolysis typically takes place during ensiling, and it averaged 5% in a data set of Huhtanen et al. [21] comparing 52 pairs of grasses and subsequent formic-acid treated silages. Thus, ensiling could be anticipated to facilitate the release of liquid from the plant cell contents. There are relatively few direct comparisons of fresh and ensiled grass in a biorefinery process, but Ayanfe et al. [22] showed that this was indeed the case (Fig. 3). There was also an interaction, so the effect of ensiling was greater when a less efficient liquid separation method was used. Unfortunately, Ayanfe et al. [22] did not analyse in detail the composition of press cake and press juice from fresh and ensiled grasses, and information of only liquid yield and DM, ash and CP concentrations in press juice are available. As liquid yield and liquid CP concentration were higher in ensiled rather than fresh grass [22], it can be deduced that press cake from silage would be drier and have lower CP concentration than that from fresh grass.

Although drying of grass in haymaking has been traditionally used as a forage preservation method, ensiling currently dominates [20]. However, grass is often partially dried by field wilting prior to ensiling. Decreased water activity prevents microbial growth and facilitates logistics due to reduced weight of the material. For extraction of solubles, dry material needs to be rehydrated, but according to Ayanfe et al. [22], drying and rehydrating resulted in similar CP extraction rate as from fresh or frozen grass (Fig. 3). Drying of grass is weather-dependent, but there are large-scale driers for forage that reduce the weather risk, although with increased costs.

Freezing is also a potential way to preserve biomass for further processing but would be economically viable only for high added value products. Freezing and thawing breaks plant cells, which may affect the ease of liquid release (Fig. 3). Preventing time lags at both freezing and thawing phases are important to prevent quality losses.

Heat or acid precipitation have been used to extract the protein from fresh grass juice, but they do not work for acidic juice from a fermented feedstock. Attempts have been made to use membrane filtration or ion exchange device to separate end products from the silage press juice, but this has proven challenging due to the high concentrations of variable components [23]. Thang and Novalin [24] reported a level of purification of lactic acid separation that was suitable only for low added value applications such as animal feed and de-icing agent.

Use of green protein directly for human consumption is a tempting idea, but so far, e.g., within European Union, protein extracted from fresh or ensiled grass has not received the novel food authorisation and cannot be commercially used [18]. It would be more likely that potential human food applications would be based on fresh grass rather than ensiled material, but there is a lot of scope to use the protein from ensiled materials in feed applications. Development of feed applications is also easier, because the end-users have less prejudices against novel products and are less demanding regarding the colour, texture and even taste of them.

### Silage characteristics affecting the biorefining process

#### Dry matter content

Typically, the first step in a green biorefinery process is a mechanical separation of liquid and solid fractions, i.e., press juice and press cake, using some type of screw press. An important attribute affecting the press juice yield is the DM concentration of the biomass. Franco et al. [12] conducted a meta-analysis, where silage characteristics were used to predict liquid yield, and silage DM concentration was the single most important factor affecting it, although relationships varied depending on the efficacy of the screw press. In their material, four different presses were used, and the liquid yields were 0.28, 0.26, 0.54 and 0.56, when adjusted to a silage DM concentration of 250 g/kg. This value could be a good target for silage DM for liquid-solid separation, as spontaneous in-silo effluent losses are minimized [25] and risks of poor fermentation reduced compared to materials with even lower DM concentration [19]. It was also clear that silage DM affected the DM concentration of the liquid—the lower the silage DM concentration, the lower also the DM concentration of the press juice [12].

In typical grass silage production, grass is wilted in the field to reduce the amount of water both for logistic and fermentation quality reasons. As an example, the average DM concentration of silages harvested in Finland is around 350 g/kg [26], but the range is large from around 200 g/kg in direct cut grass and gradually increasing depending on the extent of wilting to dry hay (850 g/kg). Typically, very little fermentation happens in forage with a DM content above 500–550 g/kg and material above this limit can be called haylage rather than silage.

If a high DM forage is used, and soluble cell content material should be separated from the cell walls, an option could be to add water or recycled press juice from the process to increase the moisture content and “wash” the solubles into the press juice. This could be a viable option as in the experiment of Ayanfe et al. [22], rehydrated dried grass (hay) resulted in similar extracted CP yields as fresh, frozen and ensiled materials when screw-pressed. The potential drawback of using wilted or dried grass could be the proteolysis that takes place during wilting/drying, but this may be less consequential than the benefits.

The risks related to ensiling of low DM biomass such as high losses in terms of effluent [25], gaseous fermentation losses as well as poor fermentation quality are well known [19]. Effluent losses form an environmental hazard if ending up in the soil or particularly in the water courses, so that collection of effluents from silos needs to be arranged. The collected effluent can also be utilized and currently used options include spraying it into fields as liquid fertilizer, diverting it to an anaerobic digester for biogas production, or feeding it to cows [27]. The spontaneously formed effluent could also be used for similar value-added purposes as the press juice separated in a biorefinery process. Spontaneous effluent is however easily perishable and should be used promptly, and the process is not controlled as in a properly functioning green biorefinery.

#### Fermentation quality

Ensiling can be considered as a pre-treatment for the biorefinery process that can be modified by strategic management options. Rinne et al. [28] used fibrolytic enzymes in ensiling regrowth grass and found both improved fermentation quality and higher press juice yield of silages with higher application levels of fibrolytic enzymes. In another study [29], no benefits in either fermentation quality or press juice yield were observed in response to fibrolytic enzyme application, but silages were made in big bales during late autumn so that low outdoor storage temperature may have limited the activity of the enzymes.

Organic acid-based additives are known to hydrolyse plant cell walls and increase the spontaneous effluent losses immediately after ensiling [25]. Several experiments have been conducted where formic acid treated silages were compared with control and inoculated silages in liquid-solid separation. The acid treatment improved fermentation quality and increased press juice yield, but the CP concentration of the press juice was lower than in the other silages so that CP captured in the press juice was lower than in the other treatment [22], but the formic acid additive treatment effect did not reach significance in the meta-analysis of Franco et al. [12]. The explanation of formic acid reducing press juice CP concentration can be in the lower solubility (less degradation) and/or acid precipitation of CP in the formic acid treated silages [22].

The optimal silage fermentation quality varies depending on the type of products targeted. Silage can be used in processes where intermediate platform chemicals such as lactic acid or volatile fatty acids are harvested, and high amounts of the target compounds should be achieved in the feedstock. To do that, Haag et al. [30] used carbonated lime to buffer the pH decline and increase lactic acid production in grass silage. An interesting example was reported by Steinbrenner et al. [31], who produced silage with maximal butyric acid concentration by adding carbonated lime and water and to extract butyric acid for industrial use. In traditional silage research for livestock use, limitation of butyric acid production has been one of the main goals.

#### Protein quality

Often protein is one of the main products of the green biorefinery process. The grass protein consists of chloroplast (green) and cytoplasma (white) proteins the majority being Rubisco enzyme. Grass protein has a good amino acid (AA) profile regarding the nutritional value, but during ensilage, protein degradation happens to a variable extent. The plant proteases are responsible for the initial degradation of protein and their activity is reduced by increased acidity that can be promoted e.g., by the use of efficient additives such as formic acid [32]. Protein degradation continues by microbial activity during the fermentation, and is positively correlated with the extent of fermentation, and can be particularly high in silages with high clostridial activity. Thus, good ensiling management practices help restrict protein degradation and ammonia formation in the silage.

When green biomass is ensiled, variable and sometimes very high concentrations of ammonia nitrogen (N) can be formed. For monogastric nutrition, the fate of AA during preservation and processing is crucial. Monogastric farm animals cannot utilize ammonia N, and it results in reduced N use efficiency and increased N excretion into manure. However, peptides or AA can be readily absorbed and metabolized by monogastric animals. In feed applications, the degradation of proteins into peptides or AA can be accepted as they can still be absorbed and metabolized by the animals. The changes in functional properties and taste caused by protein degradation are not a problem in a similar way as in food applications. According to Wilkinson [33], silages can be classified to ‘Excellent’ (ammonium-N < 50 g/kg N), ‘Good’ (ammonium-N of 50–100 g/kg N), ‘Moderate’ (ammonium-N of 100–150 g/kg N) and ‘Poor’ (ammonium-N above 150 g/kg N). Thus, in well preserved silage, the proportion of ammonia N is less than 50 g/kg N, leaving more than 950 g/kg of N nutritionally valuable demonstrating that losses in protein quality due to ensilage may not be high. However, under poor conditions, the proportion of ammonia N may be much higher emphasizing the importance of proper ensiling management practices.

**Fig. 4:**
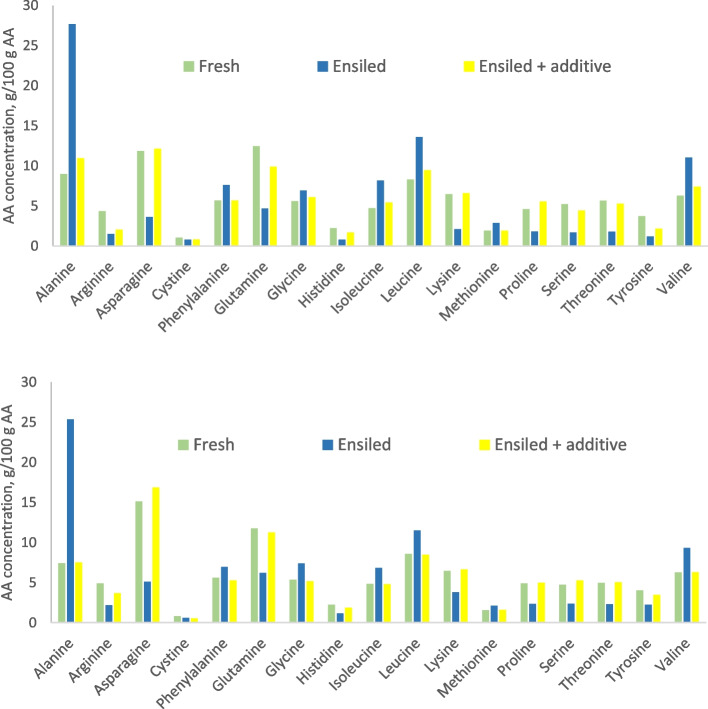
Amino acid (AA) profiles of press juices from fresh and ensiled (without an additive or with formic acid application) timothy (*Phleum pratense*; top) and red clover (*Trifolium pratensis*; bottom) (Rinne et al., unpublished)

Figure 4 shows comparison of the AA profile of press juice from fresh and ensiled timothy and red clover materials. Exceptionally low DM herbage was used (DM concentration was 118 g/kg for timothy and 110 g/kg for red clover) and it was ensiled either without any additive or using a formic acid-based additive (for details of the experiment, see [34]). In this case, ensiling without additive resulted in a very poor fermentation quality, that could be alleviated by formic acid addition (pH 4.78 vs. 4.07 and ammonia N 99 and 51 g/kg N in control vs. formic acid treated silage, respectively). The changes in fermentation quality were clearly reflected in the AA profile, as the profile of fresh and ensiled formic acid treated material was very similar, while that of poorly preserved untreated silage showed a different pattern with elevated alanine, leucine and valine proportions while those of other AA declined. The pattern was similar in timothy and red clover (Fig. 4A and B). The proportions of amino-N in total N in fresh, ensiled untreated and ensiled formic acid treated timothy were 0.73, 0.53 and 0.75, and respective values for red clover were 0.78, 0.63 and 0.82 indicating extensive amino-N degradation during ensiling in untreated silage, which could be prevented by additive application.

One challenge in using ensiled material is the separation of CP from the rest of the components in the press juice. If the press juice could be used as such without further steps, this problem would be overcome. Tampio et al. [35] and Keto et al. [36] demonstrated a system, where press juice from grass silage was used directly in the liquid feed of growing pigs with equal growth and carcass quality results as the control group with conventional liquid feed components. The press juice contained all solubles from the silage and had a CP content of 279 g/kg DM and potassium content of 71 g/kg DM. Potassium can cause diarrhoea in pigs if very high amounts are given and it may be a limiting factor in such systems. The water soluble carbohydrates and fermentation acids were also included in the press juice, and their ratio depends on the extent of fermentation. They can however be considered as useful nutrients and the organic acids have been hypothesized to have positive effects on the liquid feed stability and the gut health of pigs, although such effects could not be proven by Keto et al. [36], when also control pigs had a very good health status. However, in a laboratory trial, silage juice inhibited the growth of harmful bacteria (*Salmonella enterica* serovar Typhimurium and *Escherichia coli*; H.-L. Alakomi et al., unpublished) potentially due to the relatively high concentrations of organic acids it contained.

The press juice could potentially be used as a feed for ruminants as well. Randby [27] reported positive results from using spontaneously produced silage effluent as a feed component for dairy cows, and Rinne et al. [37] demonstrated that silage juice was readily consumed by cows. Part of the protein provided in the liquid form could potentially escape rumen degradation and the fill effect of liquid feed would probably be low. Thus, such a feeding strategy could provide a means to increase the use of locally produced forage-based feed components in the diets of high yielding dairy cows.

When assessing the quality of protein extracted from green biomass, it is important to realize that CP, i.e., total N multiplied by the constant factor 6.25 representing the N concentration in protein, can severely overestimate the actual amino-N in the fractions. An important non-amino-N nitrogenous compound in fresh green biomass is nitrate. In situations where plenty of N is available in the soil, but some factor (such as lack of radiation or water) restricts the conversion of it into plant proteins, nitrate may accumulate into plants in high amounts. Jaakkola et al. [38] demonstrated that with increasing N fertilization from 0 to 150 kg N/ha, the nitrate-N concentration in grass increased from < 0.2 to 4.8 g/kg DM. The total CP content of the highest fertilizer level grass was 170 g/kg DM, but when corrected for the nitrate-N with no nutritional value for monogastrics, it declined to 140 g/kg DM (reduction of 18%).

### Stability of fractions from the biorefinery process

The length of the aerobic stability of the feedstock and the biorefined products is an important factor affecting the timeliness costs and losses of the biorefinery. Fresh grass as well as the press juice and press cake of it are highly perishable and must be processed or preserved within hours rather than days. When ensiled, silage can typically be preserved until the harvest of the following year, and potentially even for several years. However, when exposed to air, it eventually loses the stability, and indeed, a lot of work has been dedicated to factors affecting the aerobic stability of silages [39, 40].

Rinne et al. [41] compared the aerobic stability of intact silages and press cakes from the same silages and found that extraction of liquid decreased it (90 vs. 73 h for intact silage and press cake, respectively). The stability of formic acid treated silages and press cakes (104 h) was longer than that of control and enzyme-treated silages (70 h), which did not differ from each other [41]. Similarly, Stefański et al. [42] noted faster spoilage of press cake compared to intact silage, and TMR’s prepared from them. The efficacy of TMR stabilizers (formic and propionic acids) was also more efficient on feed mixtures prepared from silage rather than press cake [42]. The factors affecting grass silage stability seem to apply similarly to press cake, and care should be taken to rapidly utilise the press cake to prevent aerobic spoilage. The faster heating of press cake compared to intact silage can be explained by the aeration during the extraction process as well as increased DM alleviating oxygen ingress and decreased organic acid concentrations that are lost in the press juice.

Fermentation end-products are water soluble so that they concentrate in the press juice. It is noteworthy that if formic acid or some other chemical additives have been applied, significant amounts of them will end up in the press juice. This will contribute to the stability of the press juice but may, on the other hand, decrease that of the press cake.

If fresh grass is used as the feedstock, the separated press juice readily ferments, and the acidification by fermentation can be used to precipitate the protein [43]. This process results in a fraction called brown juice which contains the soluble components form the grass after true protein precipitation, and where sugars have been fermented to lactic and other organic acids. This acidic liquid was successfully used as an additive for ensiled straw [44]. In an unpublished study (Rinne et al.), additional lactic acid formation and slight decline in pH (from 4.2 to 4.0 in 2 weeks) happened also in the silage juice, when it was kept in open containers in room temperature.

### Use of ensiled fibre fraction

In the case of an operation using fresh grass, a viable option for the press cake is to ensile it after liquid removal. If ensiled grass is used in the process, the press cake could even be re-ensiled after liquid removal, if it is not immediately used for processing. The quality requirements of the press cake depend on the purpose it is used for. After mechanical pressing, typically still significant amounts of moisture, soluble carbohydrates/fermentation acids and CP is left in it, which may be a benefit (feed use) or a problem (e.g. pulping for separation of pure fibres) depending on the case.

A practical use for the by-products from the biorefinery is for energy production in biogas plants utilizing anaerobic digestion [45, 46]. Vainio et al. [47] compared the biomethane production potential of original silage and the press cake, and for press cake it was 88% of that in intact silage per kg DM, obviously due to loss of highly digestible material in the press juice. The press cake could however have positive effects on biogas production when mixed e.g., with pig slurry, which would warrant further study.

The press cake is also a suitable feed material for ruminants. Trials with dairy cows have shown that it has a reasonably good feeding value [48–51], and can replace at least part of the forage proportion of even high yielding dairy cows. When soluble N is removed from the material, N use efficiency in milk production is improved. Press cake could be particularly suitable for animal groups with lower nutritional requirements, such as replacement heifers and dry cows, and also for other herbivore species such as sheep, goats and equines, but limited experimental evidence is available regarding them.

Of the above-mentioned feeding experiments, Damborg et al. [51], Sousa et al. [49] and Serra et al. [50] pressed fresh grass and ensiled the cake, while Savonen et al. [48] used ensiled grass. When fresh grass is pressed, a significant part of water soluble carbohydrates that would be used as substrate for lactic acid production during the silage fermentation process are removed, which could compromise the preservation. However, at the same time, CP and minerals with high buffering capacity are removed and the DM concentration of the mass increases which contribute positively to the ensilability. In line with these factors, the preservation quality of press cake was good as reported by Sousa et al. [49] when comparing the same grass material ensiled as intact or after screw pressing. Although the concentrations of fermentation acids were about half in screw-pressed material compared to intact grass (lactic acid 54 vs. 115 and acetic acid 12 vs. 23 g/kg DM), the pH values were close to each other and indicated good preservation quality of both of them (3.97 vs. 3.93).

During mechanical pressing, the fibre is fractured, which might benefit the microbial colonization and subsequent fibre digestion in the rumen, when used as a feed. This was suggested as a factor that could have promoted the good milk production responses observed by Damborg et al. [51] when press cake compared to intact silage was used in dairy cow diets. This effect could be specific based on the degree of fibre treatment as no effect was detected when intact and pressed fibre fraction was compared in an in vitro rumen fermentation system [52].

The grass polysaccharides can also be used as sources of fibre for various applications or hydrolysed for a variation of industrial uses. Pulping grass fibre can successfully be done [53, 54] and it requires less effort than when woody materials are used. Pihlajaniemi et al. [55] demonstrated the use of sugars from ensiled grass fibre as a feedstock for single-cell protein growth, but once the sugars have been produced, they can be used for any process. The economics may however be a constraint.

## Conclusions

Ensiled green biomass differs clearly from the fresh material but would provide many benefits in a green biorefinery operation, such as a constant year-around supply of feedstock and increased stability of the press juice and press cake. The solutions would need to be tailored, based on the particular case, due to the numerous approaches available for business concepts. Technically most steps are already solved, although more applications using ensiled rather than fresh grass would be welcome to be able to utilize the benefits of a more stable feedstock and fractions produced. The crucial point for the future of green biorefineries is to create functional concepts that can be multiplied, and to make them economically viable. It is also difficult to place monetary value to e.g., ecosystem services provided by grasslands, or to security of supply of local decentralized ways to produce feed, food, materials and energy, which may be very much needed if unexpected crises interfere with conventional supply chains (e.g., changing climate, pandemics, or political instability).

## Data Availability

Not applicable.

## Abbreviations

AA: Amino acid
CP: Crude protein
DM: Dry matter
N: Nitrogen
